# Autologous platelet-rich plasma versus hyaluronic acid, corticosteroids or saline for knee osteoarthritis: can blood draw volume serve as a proxy for platelet dose? A systematic review and meta-analysis

**DOI:** 10.1007/s00264-026-06782-7

**Published:** 2026-03-21

**Authors:** Christopher J. Centeno, Dustin R. Berger, Andrew J. Pelle, Ehren Dodson, Philippe Hernigou, Matthew B. Murphy

**Affiliations:** 1 Centeno-Schultz Clinic, Broomfield, USA; 2 Regenexx, Broomfield, USA; 3 Paris-Est Créteil University, Créteil, France; 4 Hôpitaux Universitaires Henri-Mondor, Créteil, France

**Keywords:** Platelet-rich plasma, Knee osteoarthritis, Meta-analysis, Platelet dose, Blood volume, Regenerative orthopaedics

## Abstract

**Purpose:**

To compare platelet-rich plasma (PRP) with hyaluronic acid (HA), corticosteroid (CS), or saline placebo (NS) for symptomatic knee osteoarthritis (OA) and to assess whether total blood-draw volume, a proxy for platelet dose, is associated with treatment effect.

**Methods:**

Following PRISMA, randomized controlled trials comparing intra-articular PRP with HA, CS, or NS were identified. Random-effects meta-analyses estimated mean differences (MDs) in pain (VAS) and function (WOMAC) at six and twelve months. Risk of bias was assessed with RoB 2.0 and certainty of evidence with GRADE. Subgroup analyses stratified PRP vs HA trials by total blood draw volume (< 40 mL vs ≥ 40 mL).

**Results:**

Sixty-two trials (*n* = 4,969) were included. PRP improved VAS and WOMAC versus HA, CS, and NS at six months and remained superior versus HA and CS at twelve months (insufficient studies for twelve-month PRP vs NS). In PRP vs HA trials, blood draw volume ≥ 40 mL was associated with larger improvements in six-month WOMAC (*P* = 0.004) and twelve-month VAS (*P* = 0.029). Heterogeneity was substantial (*I*^2^ > 90% for most analyses), and evidence certainty ranged from moderate to very low.

**Conclusion:**

PRP provides superior patient-reported pain and function outcomes compared with HA, CS, and NS through six months, with benefits maintained to twelve months versus HA and CS in longer-term trials. Blood-draw volume may be a useful alternate when platelet dose is unreported.

**Supplementary Information:**

The online version contains supplementary material available at 10.1007/s00264-026-06782-7.

## Introduction

Knee osteoarthritis (OA) is a prevalent joint condition characterized by the progressive degeneration of intra-articular tissues resulting in pain and loss of function. Knee OA impacts hundreds of millions of individuals globally, increasing in prevalence and severity with age [1]. Different treatment options for knee OA are available, ranging from conservative (physical therapy, weight loss, pharmacological management) [2] to surgical (debridement, cartilage repair, arthroplasty) [3]. Intra-articular injections of hyaluronic acid (HA) or corticosteroids (CS) may offer a minimally invasive means to improve symptoms associated with knee OA, and interest has grown in using autologous platelet-rich plasma (PRP). Derived from the patients’ own blood, PRP is commonly prepared by way of centrifugation to increase the concentration of platelets beyond physiological levels. Platelet-derived growth factors, delivered by way of intra-articular PRP injection, are thought to modulate the diseased microenvironment of knee OA via anti-inflammatory and anabolic effects [4]. In the context of OA, PRP may stimulate chondrocyte proliferation, enhance extracellular matrix synthesis, reduce pro-inflammatory cytokines, such as IL-1β and TNF-α, and increase hyaluronic acid secretion by synoviocytes, contributing to improved cartilage homeostasis [5]. Yet, the current clinical evidence is mixed. Multiple randomized controlled trials (RCTs) have been conducted comparing PRP to other injectates, including CS, HA, and normal saline as placebo (NS), for knee OA, with some concluding PRP to significantly improve outcomes [6–9] and others reporting PRP to fare no better than control [10–13].

Presently, there is a lack of consensus on the optimal compositional underpinnings of PRP. Robert E. Marx defined PRP as a volume of autologous plasma with a platelet concentration above baseline levels and considered effective at a concentration of one million platelets per microliter in a five-milliliter volume (5 billion platelets total) [14]. Alternatively, the Food and Drug Administration (FDA) defines PRP as autologous blood collected via a single, uninterrupted venipuncture, centrifuged to achieve a platelet concentration of at least 250,000 platelets per microliter, representing a substantially lower threshold than Marx’s definition [15]. Compositional differences in PRP preparations and the number of injections used across studies likely contribute to the reported variability in study results, yet, several recent systematic reviews have shown the overall PRP dose, or the total number of platelets administered, to be related to the magnitude of improvement reported by patients [16–18]. A complicating issue, however, is the poor adherence of many RCTs to the minimum information for studies evaluating biologics in orthopaedics (MIBO) guidelines [19], with incomplete PRP characterization limiting the number of studies available for dose-focused analyses.

One approach to extending dose-focused reviews is to leverage the blood draw volume as an indicator of the number of platelets available for capture during PRP preparation, providing a reasonable alternative for platelet dose when characterization details go unreported. Critics of PRP often cite inconsistent reporting of platelet dose as a key contributor to between-study heterogeneity and uncertainty in pooled estimates. Accordingly, this systematic review and meta-analysis evaluated the comparative efficacy of intra-articular PRP vs HA, CS, or NS for symptomatic knee OA and explored whether total blood draw volume is associated with patient-reported pain and function outcomes.

## Materials and methods

### Study inclusion/exclusion criteria

Eligible studies were level I or II randomized controlled trials (RCTs) conducted in hospital or outpatient settings that compared intra-articular autologous PRP to alternative injectates or placebo for the treatment of knee OA. The patient population included adults (> 18 years), without upper age or gender restrictions, with unilateral or bilateral mild to severe knee OA (Kellgren-Lawrence grade I-IV or equivalent). No restrictions were placed on the PRP protocol (number of injections, leukocyte content, platelet activation, blood draw volume, or injected PRP volume). Comparators included intra-articular injection(s) of HA, CS, or NS, without restrictions on the number or volume of injections.

Primary outcomes were the Western Ontario and McMaster Universities Osteoarthritis Index (WOMAC) total score (function) and the Visual Analog Scale (VAS) score (pain) at six months (or 24 weeks) following treatment. Secondary outcomes were WOMAC and VAS scores at twelve months. The outcomes were selected for their widespread use in the PRP literature. WOMAC is a multidimensional assessment for knee OA consisting of twenty-four items across pain, stiffness, and physical function [20]. Subscale scores are summed to yield a total score ranging from 0 to 96, with higher total scores indicating worse symptoms. VAS is a reliable and valid pain measure consisting of a horizontal line, 10 cm in length, anchored by ‘no pain’ and ‘worst imaginable pain’ [21]. Patients mark their pain intensity on the line, which is measured to obtain a score.

Exclusion criteria included non-randomized study designs; follow-up periods shorter than six months (24 weeks); comparisons against non-injection based treatments or injectates other than HA, CS, or NS; outcomes other than pain (VAS) and/or function (WOMAC); failure to report starting blood volume and/or prepared PRP volume; protocols that remove or destroy platelets (freeze–thaw cycles, post-activation clot removal); and non-English language publications.

### Information sources and search strategy

Literature searches were performed on March 25, 2025, using the following reference databases: Crossref, Google Scholar, PubMed, Semantic Scholar, and Scopus. Databases were searched using the following string: (Platelet-rich Plasma OR PRP) AND (Hyaluronic Acid OR Corticosteroid OR Placebo OR Saline) AND (Knee Osteoarthritis OR Gonarthrosis) AND (Randomized OR Randomized Controlled Trial). The search string was modified for reference databases that do not support Boolean operators.

### Review management and data extraction

The Evidence for Policy and Practice Information and Co-ordinating Centre Reviewer application (EPPI Reviewer 6; version 6.16.1.0) was used for systematic review management and data extraction. In accordance with the Preferred Reporting Items for Systematic Reviews and Meta-Analyses (PRISMA) guidelines [22], studies were screened against pre-established eligibility criteria. After duplicate removal, title and abstract screening was performed by one reviewer and confirmed by a second to identify RCTs investigating intra-articular PRP for knee OA. Full-text review was performed independently by two reviewers to determine study inclusion/exclusion, with disagreements resolved by a third reviewer.

Study data (demographics, methods and intervention details, and patient-reported outcomes) were extracted by one reviewer and verified by a second reviewer. When necessary, data presented in graphical format were estimated by digitizing plotted values (Plot Digitizer version 2.6.11). Means and standard deviations were derived for studies reporting alternative summary statistics (mean and range, median [IQR]) using established conversion methods (meta-converter.com) [23]. For studies with multiple PRP treatment arms (leukocyte-rich vs leukocyte-poor, or single- vs multi-injection protocols), groups were combined and outcomes were averaged to avoid multiple comparisons with a single control group. For studies with multiple control groups, HA was selected as the primary comparator based on evidence for long-term relief [24]. The blood draw volume was adjusted to account for multiple PRP injections and/or PRP volume used for laboratory characterization or standardized injection volumes.

### Risk of bias assessment

Risk of bias was assessed using the revised Cochrane risk-of-bias tool for randomized trials (RoB 2.0), which evaluates five domains: the randomization process, deviations from intended interventions, missing outcome data, measurement of the outcome, and selection of the reported result. Domain-level judgments of low risk, some concerns, or high risk were used to determine overall study risk of bias. Assessments were performed by one reviewer and confirmed by a second reviewer.

### Meta-analysis

Study results were analyzed in R (version 4.2.3), using the metafor package. Heterogeneity was assessed with Cochran’s Q statistic and the I^2^ metric. Random-effects models were used because substantial heterogeneity was present (*P* < 0.05 and *I*^2^ > 50%). Meta-analyses estimated mean differences (MDs) with 95% confidence intervals for primary and secondary outcomes. Subgroup analyses were performed based on total blood draw volume for PRP treatment. Certainty of evidence for all outcomes was assessed using the Grading of Recommendations Assessment, Development and Evaluation (GRADE) approach.

## Results

### Literature search

**Fig. 1:**
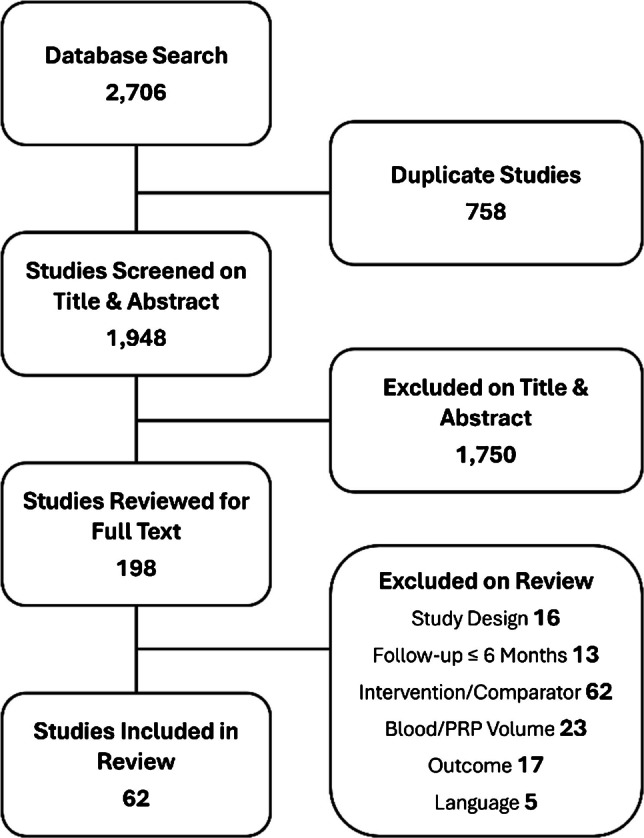
PRISMA flow diagram

A literature search of select reference databases produced a total of 2,706 studies. After removal of 920 duplicates, an additional 1,589 studies were excluded upon title and abstract screening. Full-text review was conducted on 197 studies, ultimately resulting in the inclusion of 62 RCTs for analysis [6–13, 25–78] (Fig. 1).

### Study characteristics

Together, the included RCTs reported treatment outcomes for a total of 4,969 participants, with 2,611 receiving one or more intra-articular injections of autologous PRP, and 1,321, 547, and 579 receiving one or more control injections consisting of HA, CS, and NS, respectively. The average patient age was 57.7 ± 10.0 years, with approximately 62% being female. PRP was compared with HA in 35 studies, with CS in 14 studies, and with NS in 13 studies. Two RCTs included both CS and HA control groups [42, 69], while three others included two different PRP treatment groups, focusing on PRP formulation [77], anatomical placement [68], and number of treatments, respectively. Additionally, four RCTs investigated bilateral knee OA in a total of 89 patients, with one knee randomized to the treatment group and the contralateral knee to the control group [41, 55, 65, 75].

Knee OA severity ranged from mild to severe, with 14 studies limited to mild cases and two limited to severe cases. However, most studies included patients with mild to moderate (KL II-III) knee OA. A single PRP injection was investigated in 26 studies, while two or more PRP injections were investigated in 34, with intervals of one to four weeks between treatments. Adjusted initial blood volumes ranged from eight to 150 mL and final PRP volumes ranged from two to ten mL per knee per injection. Leukocyte-poor PRP (LP-PRP) was investigated in 27 studies, while leukocyte-rich PRP (LR-PRP) was investigated in 22. One study included both PRP types, and the remaining 12 did not report leukocyte content. Approximately 25% of studies activated PRP prior to treatment with calcium. Moreover, most studies (*n* = 37) employed manual laboratory techniques to prepare PRP, while the remainder utilized a commercial PRP kit. Additional details from the included studies are provided (Tables 1, 2, 3).

**Table 1:** Characteristics of included studies comparing platelet-rich plasma to hyaluronic acid (*n* = 35). *NR* = not reported, *LP-PRP* = leukocyte poor PRP, *LR-PRP* = leukocyte rich PRP, *PRP* = undefined PRP, † = activated PRP, * = average blood/PRP volume of stated range used per knee/injection, *Man.* = manual technique, *Kit* = commercial kit, *IA* + *IO* = intra-articular + intra-osseous

Study	Patient age	% Fem	OA Grade	Control (n)	PRP (n)	Outcome of interest (Follow-Up)	Blood (mL)	PRP (mL)	Process	# of PRP Injections	Comparison to control
Abdulwahab (2024)	62.0 ± 9.9	60%	Mild to Moderate	HA (72)	PRP (72)	WOMAC (6 & 12 Month)	15	4*	Kit	1	Superior
Ahmad (2018)	56.5 ± 7.1	69%	Mild to Moderate	HA (44)	LR-PRP (45)	VAS (6 Month)	8	4	Kit	3 (Bimonthly)	Superior
Arliani (2022)	63.1 ± 5.5	83%	Mild to Moderate	HA (15)	LP-PRP (14)	WOMAC (6 Month)	15	5	Kit	3 (Weekly)	No Difference
Bansal (2021)	65.1 ± 5.2	39%	Mild to Moderate	HA (68)	LP-PRP (64)	WOMAC (6 & 12 Month)	60	8	Man	1	Superior
Buendia-Lopez (2018)	56.4 ± 2.9	52%	Mild	HA (32)	LP-PRP^†^ (33)	VAS & WOMAC (6 & 12 Month)	60	5	Man	1	Superior
Cerza (2012)	66.4 ± 10.9	56%	Mild to Moderate	HA (60)	LP-PRP (60)	WOMAC (24 Week)	12	5.5	Kit	4 (Weekly)	Superior
Cole (2017)	56.4 ± 10.4	52%	Mild to Moderate	HA (50)	LP-PRP (49)	VAS & WOMAC (24 & 52 Week)	10	4	Kit	3 (Weekly)	Superior (VAS)
Danieli (2021)	35.7 ± 7.4	24%	Mild	HA (15)	LP-PRP (14)	VAS & WOMAC (6 & 12 Month)	20	2.5	Man	3 (Weekly)	Superior
Dinesh (2020)	51.0 ± 4.6	57%	Mild	HA (30)	PRP (30)	VAS & WOMAC (24 Week)	30	5	Man	2 (Monthly)	Superior
Duymus (2017)	60.3 ± 7.3	97%	Mild to Moderate	HA (34)	LR-PRP (33)	WOMAC (6 & 12 Month)	14	3.5*	Kit	2 (Monthly)	Superior
El-Zayat (2022)	71.0 ± 4.1	48%	Severe	HA (34)	LP-PRP (33)	WOMAC (6 Month)	20	6	Man	1	Inferior
Fossati (2024)	60.5 ± 12	63%	Mild to Moderate	HA (54)	LP-PRP (54)	VAS & WOMAC (6 & 12 Month)	8	2.5*	Kit	3 (Bimonthly)	No Difference
Huang (2019)	54.7 ± 1.2	45%	Mild	HA (40)	LP-PRP (40)	VAS & WOMAC (6 & 12 Month)	8	4	Kit	1	Superior (WOMAC)
Kucukakkas (2022)	57.3 ± 10.2	75%	Mild to Moderate	HA (20)	LR-PRP (20)	VAS & WOMAC (6 Month)	50	5	Kit	1	No Difference
Li (2023)	59.2 ± 9.1	69%	Mild to Moderate	HA (33)	LR-PRP (34)	VAS & WOMAC (6 & 12 Month)	18	4	Man	3 (Weekly)	Superior
Lin (2023)	53.4 ± 3.7	73%	Mild to Moderate	HA (36)	LP-PRP (45)^†^	VAS & WOMAC (6 & 12 Month)	50	5	Man	3 (Weekly)	Superior
Louis (2018)	50.9 ± 11.7	48%	Mild to Severe	HA (17)	LP-PRP (17)^†^	VAS & WOMAC (6 Month)	34*	3	Man	1	No Difference
Malanin (2017)	46.9 ± 10.2	60%	Mild	HA (30)	LR-PRP (30)^†^	VAS (6 Month)	16	2	Man	3 (Weekly)	Superior
Ozcan (2024)	69.6 ± 12.8	75%	Mild to Moderate	HA (20)	LR-PRP (20)	VAS & WOMAC (6 Month)	10	3	Man	1	No Difference
Papalia (2016)	36.9 ± 1.1	0%	Mild	HA (24)	PRP (22)	VAS (6 & 12 Month)	8	5.5	Kit	3 (*NR*)	No Difference
Park (2021)	61.5 ± 8.9	78%	Mild to Moderate	HA (55)	LR-PRP (55)	VAS & WOMAC (6 Month)	27	3	Kit	1	No Difference
Raeissadat (2015)	58.8 ± 8.7	83%	Mild to Severe	HA (62)	LR-PRP (77)	WOMAC (24 & 52 Week)	37.5*	5*	Kit	2 (Monthly)	Superior
Raeissadat (2021)	57.0 ± 6.4	75%	Mild to Moderate	HA (49)	LR-PRP (52)	VAS & WOMAC (6 & 12 Month)	35	2	Kit	2 (3 Weeks)	Superior
Rawat (2019)	*NR*	*NR*	Mild to Moderate	HA (30)	LR-PRP (30)	VAS & WOMAC (24 Week)	20	4	Kit	1	Superior
Sathyamurthy (2022)	51.0 ± 10.2	57%	Mild to Moderate	HA (30)	PRP (30)	VAS & WOMAC (6 Month)	9*	3.5*	Man	3 (Monthly)	Superior
Shoma (2019)	51.0 ± 6.3	58%	Mild to Moderate	HA (30)	LR-PRP (30)	VAS & WOMAC (6 Month)	30	10	Man	2 (Bimonthly)	Superior
Spakova (2012)	53.0 ± 13.5	47%	Mild to Moderate	HA (60)	LR-PRP (60)	WOMAC (6 Month)	27	3	Man	3 (Weekly)	Superior
Su (2018)	52.6 ± 7.3	60%	Mild to Moderate	HA (30)	LR-PRP (25)^†^ LR-PRP IA + IO (27)^†^	VAS & WOMAC (6 & 12 Month)	38.5	6	Man	2 (Bimonthly)	Superior (IA + IO)
Szwedowski (2022)	55.3 ± 9.0	80%	Mild to Moderate	HA (24)	LP-PRP (25)	WOMAC (6 Month)	11*	7.5*	Kit	1	Superior
Vaquerizo (2013)	63.6 ± 7.2	60%	Mild to Severe	HA (42)	LP-PRP (48)^†^	WOMAC (6 & 12 Month)	36	8	Kit	3 (Bimonthly)	Superior
Varun (2018)	42.5 ± 4.6	47%	Mild	HA (30)	PRP (30)	VAS & WOMAC (24 Week)	30	5	Man	1	Superior (WOMAC)
Wang YC (2022)	62.4 ± 5.4	75%	Mild	HA (56)	LP-PRP (54)	WOMAC (6 Month)	10	4	Kit	1	No Difference
Wang Z (2022)	63.6 ± 10.5	76%	Mild to Moderate	HA (50)	LR-PRP (50)^†^	VAS & WOMAC (6 Month)	32	4	Man	3 (Weekly)	Superior (WOMAC)
Xu (2021)	57.0 ± 3.9	70%	Mild to Moderate	HA (20)	LP-PRP (30)	VAS (6 & 12 Month)	29	4	Man	3 (Bimonthly)	Superior
Yaradilmis (2020)	60.7 ± 7.9	88%	Mild to Moderate	HA (30)	LP-PRP (30) LR-PRP (30)	VAS & WOMAC (6 & 12 Month)	15	3	Man	3 (Weekly)	Superior (LR-PRP)

**Table 2:** Characteristics of included studies comparing platelet-rich plasma to corticosteroids (*n* = 14). *NR* = not reported, *LP-PRP* = leukocyte poor PRP, *LR-PRP* = leukocyte rich PRP, *PRP* = undefined PRP, † = activated PRP, * = average blood/PRP volume of stated range used per knee/injection, *Man.* = manual technique, *Kit* = commercial kit

Study	Patient age	% Fem	OA grade	Control (n)	PRP (n)	Outcome of interest (Follow-Up)	Blood (mL)	PRP (mL)	Process	# of PRP Injections	Comparison to control
Arora (2023)	54.3 ± 8.9	67%	Mild to Moderate	CS (41)	PRP (46)	VAS & WOMAC (6 Month)	37.5*	3	Man	1	Superior
Chandran (2022)	54.0 ± 6.3	55%	Mild	CS (30)	PRP (30)	VAS & WOMAC (6 & 12 Month)	30	5	Man	1	Superior
Elksnins-Finogejevs (2020)	68.3 ± 8.9	80%	Mild to Moderate	CS (17)	LP-PRP (19)	VAS (30 & 58 Week)	18	8	Kit	1	Superior
Forogh (2016)	60.1 ± 6.9	67%	Mild to Moderate	CS (16)	PRP (23)	VAS (6 Month)	20	5	Kit	1	Superior
Jubert (2017)	66.7 ± 8.0	72%	Moderate to Severe	CS (30)	LP-PRP (34)	VAS (6 Month)	40	4	Man	1	No Difference
Kumar (2021)	64.2 ± 9.2	67%	Mild to Moderate	CS (40)	LR-PRP (42)	VAS & WOMAC (6 Month)	18	4.5*	Man	1	Superior (VAS)
Longjam (2019)	50.7 ± 5.4	58%	Mild to Moderate	CS (39)	LR-PRP (38)	VAS & WOMAC (24 Week)	21.5	5	Man	2 (Bimonthly)	Superior
Malhotra (2024)	62.6 ± 10.3	16%	Mild to Moderate	CS (25)	LR-PRP (25)	WOMAC (6 & 12 Month)	20	6	Man	1	Superior
Nabi (2018)	58.8 ± 8.3	82%	Mild to Moderate	CS (34)	PRP (33)	VAS (6 Month)	50	5	Kit	3 (Monthly)	Superior
Pretorius (2022)	63.8 ± 9.7	59%	Mild to Moderate	CS (29)	LP-PRP (29)	WOMAC (6 Month)	15	5	Kit	1	No Difference
Ramesh (2019)	57.4 ± 11.1	42%	Mild	CS (131)	PRP (147)^†^	VAS & WOMAC (6 & 12 Month)	20	3.5*	Man	2 (Monthly)	Superior
Shaik (2024)	58.8 ± 8.0	58%	Mild to Moderate	CS (50)	LP-PRP (50)	VAS & WOMAC (6 & 12 Month)	20	5	Man	3 (Monthly)	Superior
Singh (2024)	60.7 ± 5.9	62%	Mild to Moderate	CS (30)	LR-PRP (30)^†^	VAS (6 Month)	20	4.5*	Man	1	Superior
Uppin (2021)	*NR*	*NR*	Mild	CS (35)	PRP (35)	VAS & WOMAC (6 Month)	40	5	Man	2 (3 Weeks)	Superior

**Table 3:** Characteristics of included studies comparing platelet-rich plasma to saline placebo (*n* = 13). *LP-PRP* = leukocyte poor PRP, *LR-PRP* = leukocyte rich PRP, *PRP* = undefined PRP, † = activated PRP, * = average blood/PRP volume of stated range used per knee/injection, *Man.* = manual technique, *Kit* = commercial kit

Study	Patient age	% Fem	OA grade	Control (n)	PRP (n)	Outcome of interest (Follow-Up)	Blood (mL)	PRP (mL)	Process	# of PRP injections	Comparison to control
Chu (2022)	54.2 ± 5.1	59%	Mild to Moderate	NS (302)	LP-PRP (308)	VAS & WOMAC (24 & 60 Week)	40	4	Man	3 (Weekly)	Superior
Dorio (2021)	64.4 ± 7.2	93%	Mild to Moderate	NS (21)	LP-PRP (20)	VAS & WOMAC (24 Week)	40	3*	Man	2 (Bimonthly)	No Difference
Elik (2020)	60.8 ± 7.4	93%	Mild to Moderate	NS (27)	LR-PRP (30)^†^	VAS & WOMAC (6 Month)	10	4	Man	3 (Weekly)	Superior
Eroglu (2017)	63.0 ± 6.4	89%	Mild to Moderate	NS (20)	LR-PRP (18)^†^	WOMAC (6 Month)	50	6	Man	3 (Weekly)	No Difference
Ghai (2019)	49.8 ± 9.4	75%	Mild	NS (20)	LP-PRP (20)^†^	VAS & WOMAC (6 Month)	55*	8	Man	1	Superior
Patel (2013)	52.8 ± 9.7	71%	Mild	NS (23)	LP-PRP (26)^†^ LP-PRP (25)^†^	VAS & WOMAC (6 Month)	50	8	Man	1 2 (3 Weeks)	Superior
Qamar (2021)	59.4 ± 4.3	63%	Mild to Severe	NS (50)	LR-PRP (50)^†^	VAS (6 Month)	20	5	Man	3 (Weekly)	Superior
Saraf (2022)	60.4 ± 7.3	55%	Severe	NS (41)	PRP (43)	VAS & WOMAC (6 Month)	38*	3	Man	3 (Monthly)	Superior
Shukla (2022)	46.8 ± 21.3	70%	Mild	NS (20)	LP-PRP (20)^†^	VAS & WOMAC (6 Month)	49*	9*	Man	1	Superior
Smith (2016)	50.1 ± 9.4	63%	Mild to Moderate	NS (15)	LP-PRP (15)	WOMAC (6 & 12 Month)	15	5.5*	Kit	3 (Weekly)	Superior
Tucker (2021)	57.4 ± 2.6	41%	Mild to Moderate	NS (6)	LP-PRP (11)	VAS (6 & 12 Month)	150	5	Kit	1	No Difference
Wu (2018)	63.3 ± 6.8	75%	Mild to Moderate	NS (20)	LR-PRP (20)	WOMAC (6 Month)	10	4	Kit	1	Superior
Yoshioka (2024)	66.9 ± 9.3	70%	Mild to Moderate	NS (14)	LP-PRP (15)	VAS & WOMAC (24 Week)	27	6	Kit	3 (Weekly)	Superior

### Risk of bias and quality assessment

**Fig. 2:**
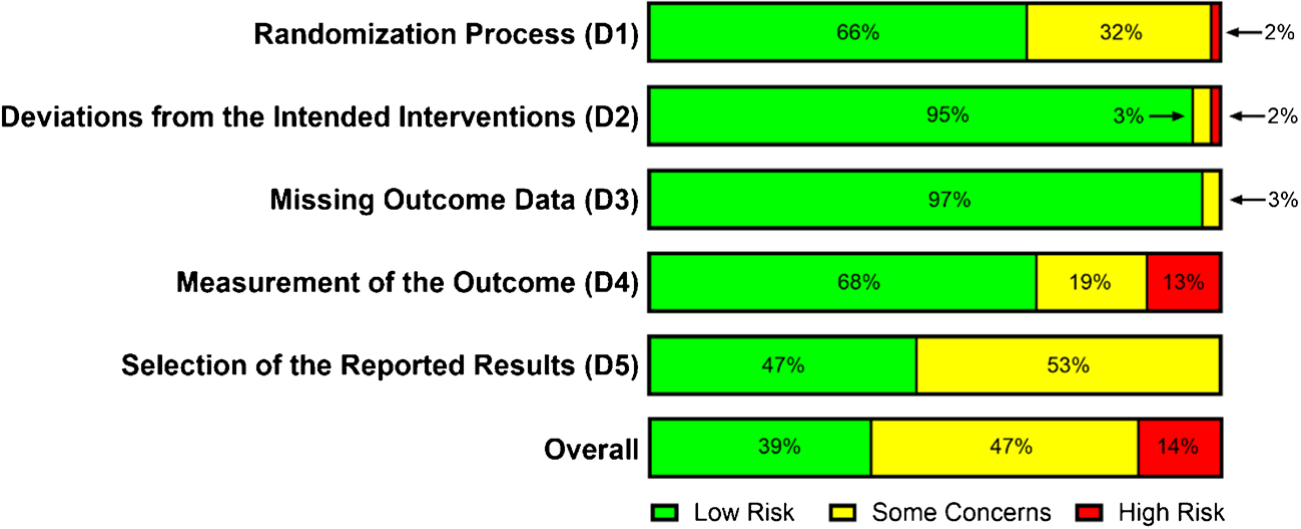
Overall risk of bias assessment (*n* = 62 studies)

Risk of bias assessment indicated that 47% of RCTs raised some concerns, 39% were judged to be at low risk of bias, and 14% were judged to be at high risk of bias (Fig. 2). Many RCTs with some concerns did not analyze data in accordance with a pre-specified analysis plan finalized prior to unblinding. Among RCTs judged to be at high risk of bias, most concerns related to outcome measurements potentially differing between intervention groups (Supplemental Fig. 1). The quality of evidence, as assessed using the GRADE approach, ranged from moderate to very low depending on the outcome, control group, and follow-up. A sensitivity analysis limited to low risk of bias studies yielded largely unchanged effect estimates. Funnel plots and related Egger’s tests did not reveal significant publication bias for most outcomes assessed. Overall, evidence certainty was primarily downgraded due to inconsistency across studies and imprecision in effect estimates (Supplemental Table).

### Primary outcomes (6-Month VAS and WOMAC)

Twenty-four studies were evaluated for the difference between PRP and HA in terms of six-month VAS pain scores, and 27 reported six-month total WOMAC scores. PRP produced statistically significant improvements in both pain (VAS *MD* = −0.72 [−1.05, −0.38], *P* < 0.001, *I*^2^ = 94.0%) and function (WOMAC MD = −5.63 [−9.09, −2.16], *P* = 0.001, *I*^2^ = 99.1%) compared with HA. Twelve and nine RCTs were evaluated for the difference between PRP and CS in terms of six-month VAS and WOMAC, respectively, demonstrating significant improvements with PRP (VAS *MD* = −1.52 [−2.04, −1.01], *P* < 0.001, *I*^2^ = 91.9%; WOMAC *MD* = −6.04 [−10.17, −1.90], *P* < 0.001, *I*^2^ = 92.9%). Ten RCTs comparing PRP with NS similarly favored PRP for both (VAS MD = −1.60 [−2.23, −0.96], *P* <  p=0.004, *I*^2^ = 91.5%; WOMAC *MD* = −10.04 [−16.12, −3.95], *P* = 0.001, *I*^2^ = 96.8%). Effect estimates were smallest in RCTs comparing PRP with HA and largest in those comparing PRP with NS placebo; however, heterogeneity remained substantial across primary outcomes (Table 4).

**Table 4:** Meta-analyses of primary outcomes

Outcome	Comparator	Studies	Participants	Mean difference [95% CI]	*I*^2^	*P* Value	GRADE
6-Month VAS	HA	24	1,651	−0.72 [−1.05, −0.38]	94.0%	< 0.001	Moderate
6-Month VAS	CS	12	1,020	−1.52 [−2.04, −1.01]	91.9%	< 0.001	Low
6-Month VAS	NS	10	1,051	−1.60 [−2.23, −0.96]	91.5%	< 0.001	Moderate
6-Month WOMAC	HA	27	2,120	−5.63 [−9.09, −2.16]	99.1%	0.001	Low
6-Month WOMAC	CS	9	833	−6.04 [−10.17, −1.90]	92.9%	0.004	Very Low
6-Month WOMAC	NS	10	949	−10.04 [−16.12, −3.95]	96.8%	0.001	Low

### Secondary outcomes (12-Month VAS and WOMAC)

Thirteen RCTs were evaluated for the difference between PRP and HA at the twelve-month follow-up, with statistically significant improvements in both VAS (*MD* = −1.27 [−1.77, −0.77], *P* < 0.001, *I*^2^ = 91.0%) and WOMAC (MD = −9.86 [−12.82, −6.91], *P* < 0.001, *I*^2^ = 94.2%) scores reported by those receiving PRP. Similarly, four RCTs were evaluated for the difference between PRP and CS, with both VAS (*MD* = −2.99 [−3.21, −2.78], *P* < 0.001, *I*^2^ = 0%) and WOMAC (*MD* = −17.45 [−22.63, −12.26], *P* < 0.001, *I*^2^ = 86.1%) favoring PRP. Effect estimates for VAS and WOMAC were smaller among studies comparing PRP to HA than those comparing PRP to CS. Both outcomes’ effect estimates were larger at twelve months than at six months. A wide range of heterogeneity (*I*^2^ = 0% to 94.2%) was observed among secondary outcomes (Table 5). An insufficient number of studies (*n* = 2) were available to meaningfully contribute to a meta-analysis of twelve-month outcomes for PRP versus NS.

**Table 5:** Meta-analyses of secondary outcomes

Outcome	Comparator	Studies	Participants	Mean difference [95% CI]	*I*^2^	*P* Value	GRADE
12-Month VAS	HA	13	966	−1.27 [−1.77, −0.77]	91.0%	< 0.001	Moderate
12-Month VAS	CS	4	474	−2.99 [−3.21, −2.78]	0%	< 0.001	Moderate
12-Month WOMAC	HA	13	1,208	−9.86 [−12.82, −6.91]	94.2%	< 0.001	Moderate
12-Month WOMAC	CS	4	488	−17.45 [−22.63, −12.26]	86.1%	< 0.001	Very Low

### Subgroup analysis on total blood volume

**Fig. 3:**
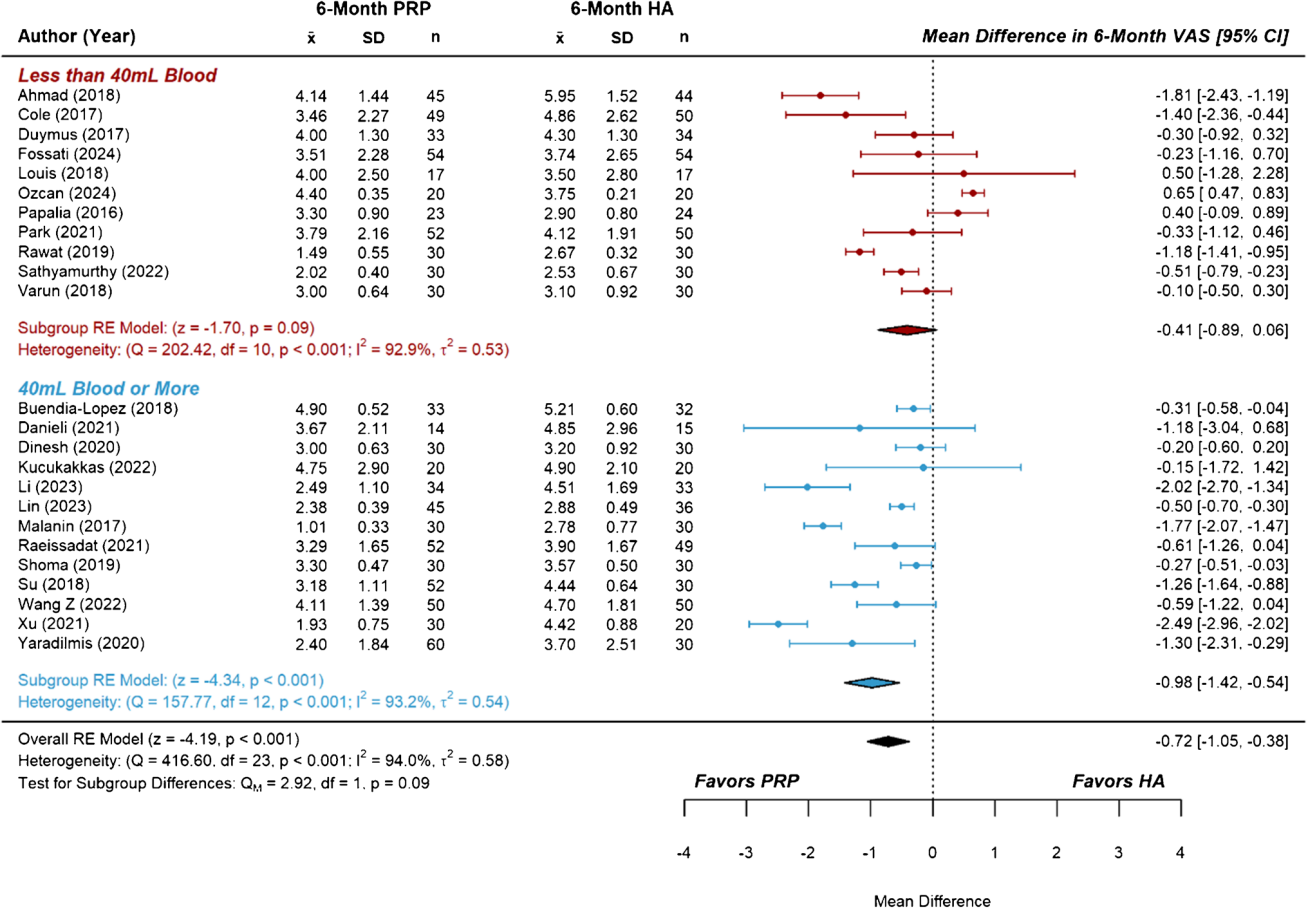
Forest plots and meta-analysis of reported six-month VAS scores from studies comparing platelet-rich plasma to hyaluronic acid (*n* = 24). Subgroups were separated based on total blood volume used to prepare one or more PRP treatments

**Fig. 4:**
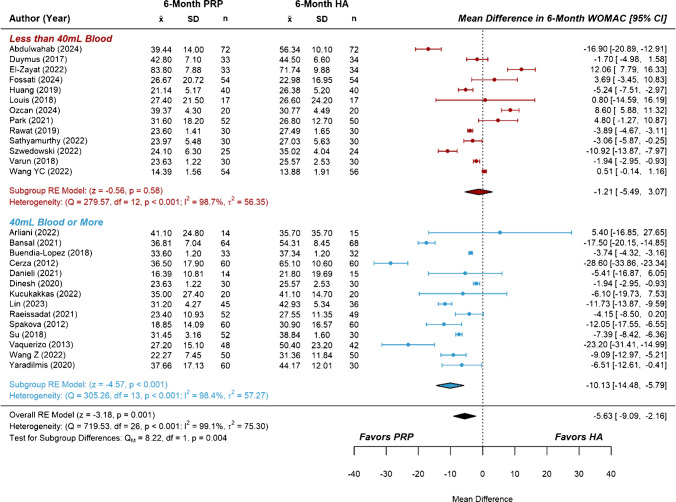
Forest plots and meta-analysis of reported six-month WOMAC scores from studies comparing platelet-rich plasma to hyaluronic acid (*n* = 27). Subgroups were separated based on total blood volumes used to prepare one or more PRP treatments

**Fig. 5:**
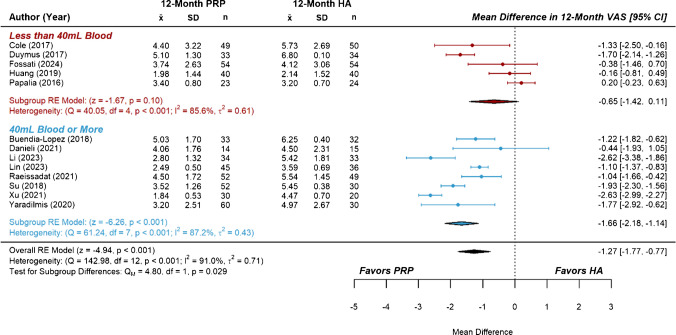
Forest plots and meta-analysis of reported twelve-month VAS scores from studies comparing platelet-rich plasma to hyaluronic acid (*n* = 13). Subgroups were separated based on total blood volumes used to prepare one or more PRP treatments

**Fig. 6:**
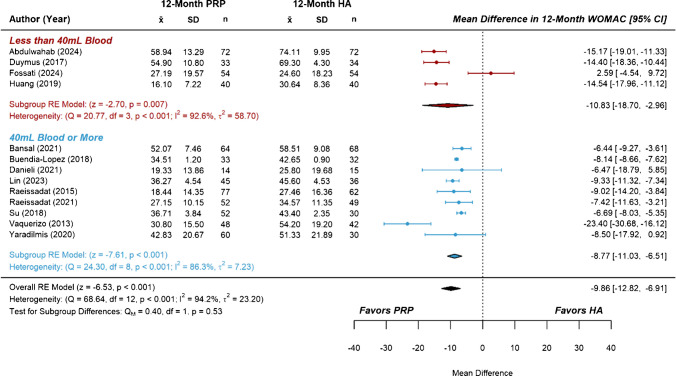
Forest plots and meta-analysis of reported twelve-month WOMAC scores from studies comparing platelet-rich plasma to hyaluronic acid (*n* = 13). Subgroups were separated based on total blood volumes used to prepare one or more PRP treatments

Studies comparing PRP with HA represented the largest subset of included RCTs, containing more than the minimum number recommended for subgroup analysis on both primary and secondary outcomes. Subgroups were separated based on the total blood volume used to prepare the course of PRP treatment(s), with a volume of 40 mL dividing the six-month data into two similarly sized groups of studies. The effect estimate for six-month VAS score from the group of studies using less than 40 mL of blood was not significant compared to HA (*MD* = −0.41 [−0.89, 0.06], *P* =  0.09, *I*^2^ = 92.9%). In contrast, the group of studies using 40 mL or more of blood demonstrated a significant effect estimate (*MD* = −0.98 [− 1.42, −0.54], *P* < 0.001, *I*^2^ = 93.2%), though a test of subgroup differences was not significant (Fig. 3). Similarly, the effect estimate for six-month WOMAC scores from the low blood volume group was not significant compared to HA (*MD* = −1.21, [−5.49, 3.07], *P* = 0.58, I^2^ = 98.7%), while that of the higher blood volume group showed significant improvement (*MD* = −10.13 [−14.48, −5.79], *P* < 0.001, *I*^2^ = 98.4%). A test of subgroup differences was significant, *P* = 0.004 (Fig. 4). At the twelve-month follow-up, the effect estimate for VAS scores from the low blood volume group was not significant compared to HA (*MD* = −0.65 [−1.42, 0.11], *P* = 0.100, *I*^2^ = 85.6%). However, the group using higher blood volumes showed significant improvement (*MD* = −1.66 [−2.18, −1.14], *P* < 0.001, *I*^2^ = 87.2%), and a test of subgroup differences was significant, *P* = 0.029 (Fig. 5). Lastly, there were no significant subgroup differences for the effect estimates of twelve-month WOMAC scores, as both the low blood volume group (*MD* = −10.83 [−18.70, −2.96] *P* = 0.007, *I*^2^ = 92.6%) and the high blood volume group (*MD* = ] -8.77 [-11.03, -6.51], *P* < 0.001, *I*^2^ = 86.3%) showed significant improvements over HA (Fig. 6). Considerable heterogeneity (*I*^2^ = 85.6% to 98.7%) remained following subgroup analysis.

## Discussion

Knee OA is a common degenerative disease that is increasing in prevalence and imposing a mounting financial burden, underscoring the need for more effective interventional therapies [79]. Minimally invasive injections, including intra-articular CS for pain relief and HA for viscosupplementation, are widely used. PRP contains an array of growth factors, cytokines, and other bioactive proteins, and is thought to modulate inflammation and support tissue repair [4]. In this systematic review and meta-analysis, PRP provided superior pain relief and functional improvement in patients with mild-to-severe symptomatic knee OA compared with HA, CS, and saline placebo. Because platelet-dose reporting is inconsistent across RCTs, between-study heterogeneity is often cited as limiting interpretability; our findings suggest that total blood volume may serve as a reasonable alternative when platelet dose is unreported. When stratified by total blood volume used for PRP preparation, trials using lower blood volumes showed outcomes comparable to HA, whereas trials using high blood volumes reported significantly improved patient outcomes.

A large proportion of studies investigating autologous PRP, including many trials in the current review, did not adequately characterize the biological composition of their preparations. Although more platelets can be obtained from larger blood volumes, without characterization, it is difficult to determine whether the therapeutic effect relates to the absolute platelet number or platelet concentration. Baseline platelet counts vary widely in whole blood (approximately 150,000 to 450,000 per microliter), with a potential three-fold difference between individuals [80]. However, this variability is expected to average out given the large number of patients included. Many commercial PRP kits also process fixed blood volumes, (typically 20 to 60 mL, but as low as eight mL), so system selection directly affects the blood volume processed and the number of platelets available for capture.

Several recent studies and reviews have suggested that the total number or concentration of platelets within PRP is related to improved patient outcomes for knee OA. Berrigan et al. conducted a systematic review that identified a potential dose–response relationship for knee OA, with higher doses showing improved outcomes for functional measures, but also noted that a lower dose may still provide some benefit [81]. Boffa et al. led a cohort study to determine if platelet concentration in PRP influenced treatment outcomes for knee OA [82]. The findings revealed that higher platelet concentrations were associated with improved clinical outcomes, including greater improvements in functional scores compared to lower concentrations, and a significantly reduced failure rate. A meta-analysis by Bensa et al. also showed high-platelet PRP to provide better pain relief and functional improvement for knee OA than low-platelet PRP [16], while another by Tao et al. concluded triple-dose PRP therapy to be significantly better than single-dose therapy for improving pain [83]. Moreover, Bansal et al. recommended an absolute count of ten billion platelets for a PRP preparation to have a sustained chondroprotective effect [6]. However, several other recent meta-analyses have suggested that higher platelet concentrations in PRP may be unnecessary or may even attenuate the desired effects [84, 85].

Our meta-analysis showed that RCTs using PRP prepared from higher blood volumes reported larger improvements in six-month VAS and WOMAC and in twelve-month VAS compared with HA, whereas trials using lower blood volumes reported outcomes more comparable to HA. If blood volume is considered an indicator of PRP yield, or the total platelets administered, this finding aligns with prior work suggesting higher overall platelet doses may be more effective for knee OA. Using typical platelet counts, 40 mL of whole blood contains approximately six to 18 billion platelets, whereas an eight mL blood tube contains roughly 1.2 to 3.6 billion platelets, a total dose considerably lower than the absolute dose of ten billion platelets recommended by Bansal et al. [6].

Quality of evidence was assessed as moderate to very low, depending on outcome, follow-up, and control group, largely because of imprecision in effect estimates and the presence of considerable heterogeneity amongst endpoints. High heterogeneity may be attributed to several factors. Firstly, the degree of OA in the included patient population varied widely across RCTs, with Buendia-Lopez et al. [29], Patel et al. [54], Uppin et al. [71], and others limiting inclusion criteria to only mild knee OA, and El-Zayat et al. [36] and Saraf et al. [61] including only those with severe knee OA. Many of the RCTs involved patients with bilateral knee OA, opting to treat the more affected knee, and a few, including those by Ghai et al. [41] and Pretorious et al. [55], required bilateral knee OA per their study design. Yet, contralateral knee pain has been reported to influence WOMAC outcomes from the primary knee [86]. The number of PRP injections, volumes injected, and duration between two or more injections varied among included RCTs. Park et al. reported outcomes following a single injection of three mL PRP [53], while Vaquerizo et al. reported outcomes following three bimonthly injections of eight mL PRP [72]. Further, an assortment of commercial PRP kits and manual laboratory techniques requiring varying blood volumes were utilized, some involving collection of a buffy coat and/or addition of an activating agent, leading to PRP preparations with differing amounts of platelets, leukocytes, and growth factors. Although subgrouping based on the total blood volume used for PRP preparation yielded significant differences in effect estimates for several endpoints when compared to HA, considerable heterogeneity persisted. Additional limitations include differences in pre- and/or post-treatment regimen, including the use of any medications or physical therapy, and the presence of expectation bias among unblinded patients that could also affect patient response. All patient outcome measures utilized subjective questionnaires to discern treatment effects and lacked objective findings such as medical imaging.

Despite the substantial heterogeneity, patients treated with autologous PRP for knee OA generally report better outcomes than those treated with comparator injections. In trials comparing PRP with HA, higher blood volumes used to prepare PRP were associated with greater improvements. Nevertheless, biological characterization of PRP remains limited across many RCTs, hindering our understanding of how platelet dose influences clinical response. Standardized reporting of PRP composition in accordance with MIBO guidelines and well-designed prospective dose–response trials are needed to better define platelet dosing standards going forward. Overall, these data suggest that total blood volume may serve as a practical stand-in when platelet dose reporting is missing.

## Supplementary Information

Below is the link to the electronic supplementary material.

Supplementary file1 (DOCX 829 KB)

## Data Availability

The data that support the findings of this study are available from the corresponding author upon reasonable request.
