# Supplementary material for: Autologous platelet-rich plasma versus hyaluronic acid, corticosteroids or saline for knee osteoarthritis: can blood draw volume serve as a proxy for platelet dose? A systematic review and meta-analysis
Source: Int Orthop. 2026 Mar 21;50(5):981–97. doi: 10.1007/s00264-026-06782-7 (PMC13179890; doi:10.1007/s00264-026-06782-7)
Supplement: Supplementary file 1 — Supplementary file1 (DOCX 829 KB) [file 264_2026_6782_MOESM1_ESM.docx]

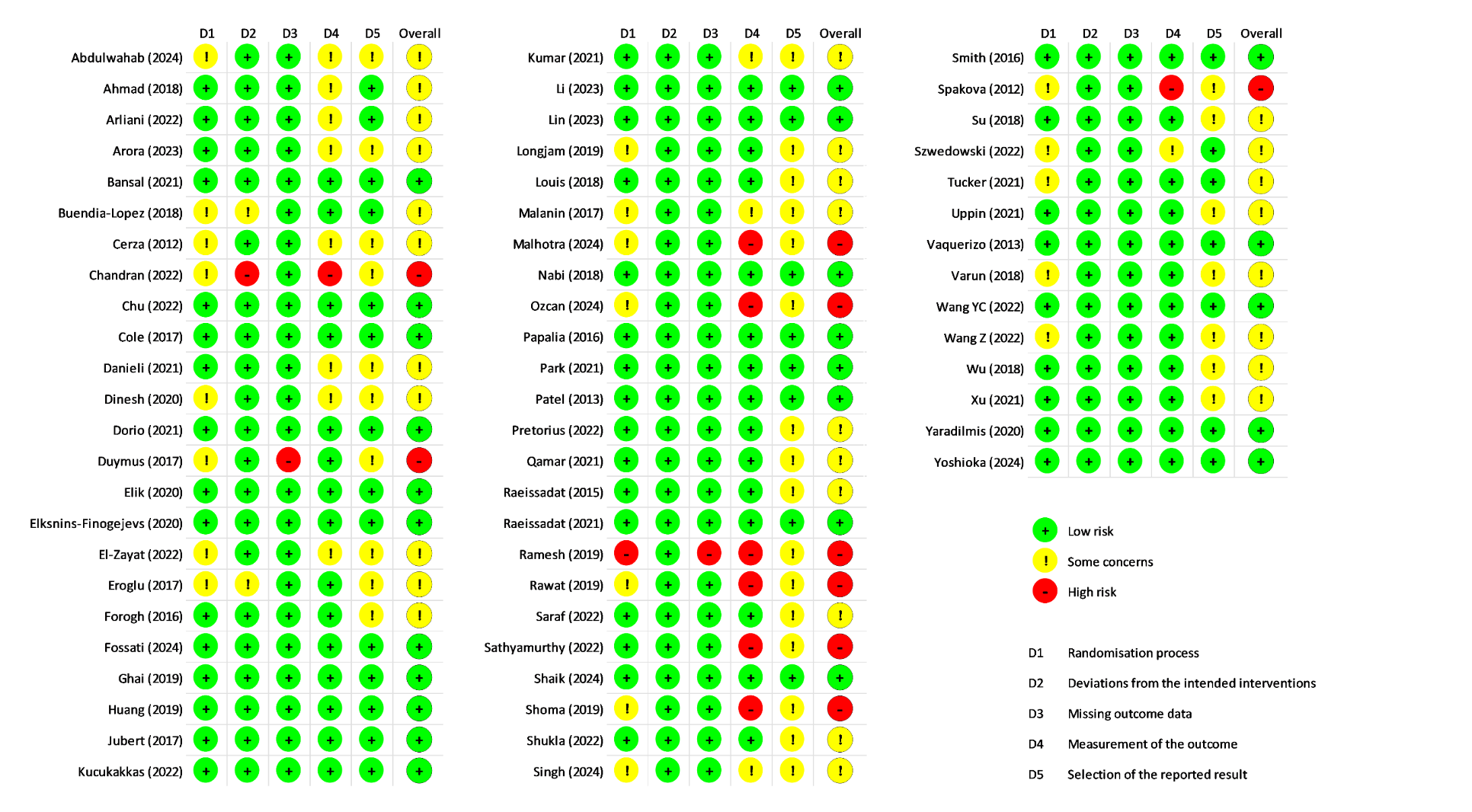


**Supplemental Fig. 1 –** Detailed risk of bias assessment (n = 62 studies).

| **Outcome Measure** | **Comparison** | **Risk of Bias** | **Inconsistency** | **Indirectness** | **Imprecision** | **Publication Bias** | **Quality** |
| --- | --- | --- | --- | --- | --- | --- | --- |
| 6-Month VAS | HA | No Serious Risk of Bias | Serious Inconsistency | No Serious Indirectness | No Serious Imprecision | Not Detected | Moderate |
| 6-Month VAS | CS | No Serious Risk of Bias | Serious Inconsistency | No Serious Indirectness | No Serious Imprecision | Serious Publication Bias | Low |
| 6-Month VAS | NS | No Serious Risk of Bias | Serious Inconsistency | No Serious Indirectness | No Serious Imprecision | Not Detected | Moderate |
| 6-Month WOMAC | HA | No Serious Risk of Bias | Serious Inconsistency | No Serious Indirectness | Serious Imprecision | Not Detected | Low |
| 6-Month WOMAC | CS | Serious Risk of Bias | Serious Inconsistency | No Serious Indirectness | Serious Imprecision | Not Detected | Very Low |
| 6-Month WOMAC | NS | No Serious Risk of Bias | Serious Inconsistency | No Serious Indirectness | Serious Imprecision | Not Detected | Low |
| 12-Month VAS | HA | No Serious Risk of Bias | Serious Inconsistency | No Serious Indirectness | No Serious Imprecision | Not Detected | Moderate |
| 12-Month VAS | CS | No Serious Risk of Bias | No Serious Inconsistency | No Serious Indirectness | Serious Imprecision | Not Detected | Moderate |
| 12-Month WOMAC | HA | No Serious Risk of Bias | Serious Inconsistency | No Serious Indirectness | No Serious Imprecision | Not Detected | Moderate |
| 12-Month WOMAC | CS | Serious Risk of Bias | Serious Inconsistency | No Serious Indirectness | Serious Imprecision | Not Detected | Very Low |

**Supplemental Table –** Level of Evidence using GRADE approach.
